# Protective Effect of Self-Compassion to Emotional Response among Students with Chronic Academic Stress

**DOI:** 10.3389/fpsyg.2016.01802

**Published:** 2016-11-22

**Authors:** Yonghong Zhang, Xi Luo, Xianwei Che, Wenjie Duan

**Affiliations:** ^1^School of Cultural and Social Development, Southwest UniversityChongqing, China; ^2^Monash Alfred Psychiatry Research Centre, Central Clinical School and the Alfred, Monash UniversityMelbourne, VIC, Australia; ^3^Department of Sociology, Wuhan UniversityWuhan, China

**Keywords:** self-compassion, chronic academic stress, stress-buffering effect, emotional response, mediation

## Abstract

The literature has shown that self-compassion is a protective factor of an individual’s emotional response to chronic stress. However, this stress-buffering effect has not been completely analyzed in individuals who report significantly high academic stress. The present study explored the role of self-compassion in a group of undergraduate students who experience chronic academic stress. A total of 208 undergraduate students who were preparing for the Postgraduate Entrance Examination (PEE) were recruited and completed the Self-Compassion Scale, Adolescent Self-Rating Life Event Check List, and Positive and Negative Affect Schedule. Differences analysis confirmed that the participants reported significantly higher academic stress than their peers who were not preparing for PEE. Self-compassion positively related to positive affect but negatively related to negative affect and learning stress. Further analysis showed that self-compassion negatively mediated the relationship between chronic academic stress and negative affect. Findings imply that self-compassion-centered interventions can be developed in the educational context to assist students cope with chronic academic stress.

## Introduction

Human beings suffer from an extensive variety of daily stressful events, such as academic failure, social embarrassment, and traumatic events. Stress is defined as a state of threatened homeostasis that is induced by the internal or external environment, thereby threatening an individual’s physical and mental health and further calling for restoration (Lazarus and Cohen, 1977). In general, stress is associated with decreased well-being, increased negative affect, and various affective disorders, such as depression and post-traumatic stress disorder (Habra et al., 2003; Hoffman and Al’Absi, 2004; Marin et al., 2011; Cheng et al., 2014). However, this case is not constant. Studies have also indicated that people with a few positive qualities can live through traumatic events, manage them well, and stay healthy (Carver and Antoni, 2004; Duan and Guo, 2015; Duan et al., 2015a). These findings are consistent with the Cognitive Transaction Model of Stress (Lazarus and Folkman, 1984). This model underscores that stress is recognized as a transactional and dynamic state that depends on the individual appraisal of the stimulus and behavioral responses to it. This model emphasizes the interactions between personal characteristics and situational factors rather than objective stressors alone (Lazarus and Folkman, 1984). Cross-sectional and longitudinal studies demonstrated that individuals with positive qualities, such as caring, inquisitiveness, and self-control, often perceived limited stress and showed an improved mental well-being (Duan et al., 2015b; Duan, 2016b). Therefore, the role of positive qualities in stressful situation should be investigated.

Recently, self-compassion has been increasingly recognized as a positive quality to facilitate mental well-being. Self-compassion is described as the disposition of accepting and caring for oneself, particularly as treating oneself with kindness and concern in adversity (Neff, 2003a,b). During the process of self-compassion, individuals are encouraged to recognize related experiences to oneself and others with metacognitive activities. Theoretically, self-compassion is a multi-dimensional construct with six basic components: self-kindness versus self-judgment, where self-kindness is being kind and careful towards oneself in dealing with stress; humanity versus isolation, where humanity means considering one’s experiences as an unavoidable component of the human experience; and mindfulness versus over-identification, in which mindfulness is holding painful thoughts and feelings in mindful awareness without avoiding or exaggerating them. Although six separate but related factors were obtained through exploratory factor analysis, an overreaching factor of self-compassion was recommended in both research and practice by the author (Neff, 2003b).

Existing studies have shown that self-compassion buffer both acute stress induced in laboratory settings (Pace et al., 2009; Bluth et al., 2015) and chronic life stress, such as childhood maltreatment and academic stress (Ying and Han, 2009; Vettese et al., 2011). For example, individuals with considerably high self-compassion showed substantially low autonomic response and anxiety when they were exposed to acute psychosocial threat (Neff et al., 2007a; Breines et al., 2014). Vettese et al. (2011) determined that self-compassion was reported to mediate the relationship between the experience of maltreatment in childhood and later emotion dysregulation. In the educational context, chronic academic stress is the most common. The literature that focuses on this topic has suggested that self-compassion could reduce the negative influence of chronic academic stress on academic performance and emotional well-being. Neff et al. (2005) determined among undergraduates who perceived their recent midterm grade as a failure that self-compassion was positively associated with mastery and perceived competence but negatively associated with performance avoidance. Other studies reported that self-compassion buffered the influence of academic stress (e.g., limited goal progress, academic burnout) on negative affect and depression in undergraduates (Kyeong, 2013; Hope, 2014). One study also determined that self-compassion was negatively associated with homesickness and depression in students who transited to college (Terry et al., 2013), thereby further reporting that self-compassion could reduce the influence of low satisfaction with social life on homesickness.

Nevertheless, the existing studies conducted in the educational context did not identify their participants as stressful individuals. The participants recruited in these studies were often undergraduate freshmen or master’s students (Ying and Han, 2009; Terry et al., 2013; Hope, 2014). Although the chronic academic stress is a typical life stressor to students, whether they were undergoing academic or other types of life stress is unclear. Accordingly, the current study aimed to analyze if self-compassion can mediate the relationship between chronic academic stress and emotional health among undergraduates who were preparing for the Postgraduate Entrance Examination (PEE). PEE is a typical and well-recognized chronic stressor for students in China. We hypothesize that undergraduates who were preparing for PEE would report higher perceived academic stress than those who were not preparing for this examination. We further assumed that self-compassion would reduce the negative effect of the perceived academic stress on emotional health among students preparing for PEE.

## Materials and Methods

### Participants and Procedures

All participants (*n* = 208; 66 males and 142 females; age *M* = 21.67, *SD* = 0.93) were recruited from libraries and study rooms in Southwest University in China. Before participating in this study, they were asked to indicate that they have already registered for the 2016 PEE and reported how long they have prepared for this examination (1 = approximately 3 months; 2 = 3–6 months; 3 = 6–12 months; 4 = over 12 months). All participants voluntarily participated in this study. Another compared sample was included to test if the participants preparing for PEE were under academic stress. This compared sample comprises 300 undergraduate students who are not preparing for PEE from the same university. None of them reported any history of neurological or psychiatric illness. The procedures of the present study were approved by Southwest University, and all the participants signed a written informed consent before the start of their participation.

### Measurements

#### Self-Compassion Scale

The 26-item Self-Compassion Scale (SCS) was adopted to measure individual differences in self-compassion and comprises six subscales: self-kindness, self-judgment, common humanity, isolation, mindfulness, and over-identification. The participants were asked to indicate how they typically act toward themselves in difficult times using a five-point Likert scale (from 1 “never” to 5 “almost always”) (Neff, 2003b). SCS showed well-established psychometric characteristics in previous studies (α = 0.93). Chen et al. (2011) reported the Cronbach’s alpha (0.83) and test–retest reliability (0.89) of the Chinese version. In the present study, the total scale reported a Cronbach’s alpha coefficient of 0.80. For the subscales, the internal consistency coefficients were between 0.60 and 0.77.

#### Adolescent Self-Rating Life Event Check List

The Adolescent Self-Rating Life Event Check List (ASLEC) uses 26 negative life events collected from multiple daily stress domains to evaluate stressful experiences within the past 12 months. These life stressors can be conceptualized into six factors, namely, interpersonal, learning stress, punishment, bereavement, health adaptation, and other factors (Liu et al., 1997). The participants report the effect of each life stressor on their lives using a 5-point Likert scale (1–5 means “not at all” to “extremely severe,” respectively). The learning stress score was calculated to measure the academic stress in this study. The internal consistency of this scale is 0.85 and test–retest reliability is 0.69 (Liu et al., 1997). The total scale demonstrated good internal consistency (α = 0.93) in the current study. For the scales of the six factors, the internal consistency coefficients ranged from 0.55 to 0.91, among which the learning stress factor scale reported a Cronbach’s alpha coefficient of 0.61.

#### Positive and Negative Affect Schedule

The participants reported how much they experienced positive and negative affect over the past months using the Positive and Negative Affect Schedule (PANAS) (Watson et al., 1988). PANAS is a 20-item scale with 10 emotion words that assess the positive affect and another 10 words that assess the negative affect. The participants rated these words from 1 to 5 (very slightly or not at all to extremely). Watson et al. (1988) reported the good psychometric properties of this scale, with the Cronbach’s alphas ranging from 0.84 to 0.87 for the negative affect and from 0.86 to 0.90 for the positive affect. The Chinese version of PANAS has a Cronbach’s alpha of 0.83 for the negative affect and 0.85 for the positive affect (Huang, 2003). The internal consistency coefficient of this scale was 0.86 for the negative affect and 0.82 for positive affect in the current study.

### Statistical Analysis

Descriptive statistics and correlation analysis were conducted using SPSS Statistics. We first compared the difference in the reported learning stress between the undergraduate students preparing and not preparing for PEE using independent-sample *t*-test. In the sample of undergraduate students preparing for PEE, correlations were calculated among the learning stress, total score of self-compassion, subscales of self-compassion, and positive and negative affects. Previous studies indicated significant gender differences of self-compassion between males and females (Neff, 2003b). Thus, partial correlation was conducted with gender as the control variable. Finally, mediation analysis was conducted with bootstrapping method using PROCESS (Hayes, 2013) among undergraduate students preparing for PEE. This method can provide confidence limits for the specific indirect effect, as well as include multiple mediators in the same model (Preacher and Hayes, 2008). The bias-corrected and accelerated (BCa) bootstrap estimates presented in the current study were based on 5,000 bootstrap samples.

## Results

### Differences Analysis on the Perceived Stress

The levels of learning stress between the two samples were compared. We calculated the learning stress (*M* = 8.52, *SD* = 3.78) in the sample of undergraduate students not preparing for the entrance examination. Independent-sample *t*-test revealed significant difference in the learning stress between the two groups (*t*_(506)_ = 2.69, *p* < 0.005). The participants preparing for PEE showed higher learning stress than the general undergraduate students. These results confirm that the participants preparing for PEE feel stressed about their learning and the coming examination.

### Descriptive and Correlations Analysis

A total of 13.46% of the students preparing for PEE have spent approximately 3 months to prepare for this examination, 35.10% indicated 3–6 months, 38.46% indicated 6–12 months, and 12.98% reported over 1 year. The Pearson correlation between the preparing time period and learning stress was negative (*r* = -0.15, *p* < 0.05). Consequently, gender and preparing time period were considered controlling variables. **Table 1** presents the descriptive statistics and correlations among all variables. After controlling for gender, age, and preparing time, learning stress was positively associated with negative affect but not associated with positive affect (see **Table 1**). Furthermore, learning stress was negatively correlated with the total score of self-compassion and was positively associated with the subscales of self-judgment, isolation, and over-identification.

**Table 1:** Correlation coefficients between learning stress, affects, self-compassion.

Variables	1	2	3	4	5	6	7	8	9	10
(1) Learning stress	_									
(2) Negative affect	0.37^∗∗^	_								
(3) Positive affect	0.04	–0.17^∗^	_							
(4) Self-compassion	-0.28^∗∗^	–0.47^∗∗^	0.40^∗∗^	_						
(5) Self-judgment	0.28^∗∗^	0.37^∗∗^	0.08	-0.58^∗∗^	_					
(6) Common humanity	-0.03	–0.08	0.40^∗∗^	0.53^∗∗^	0.09	_				
(7) Isolation	0.35^∗∗^	0.51^∗∗^	-0.12	-0.69^∗∗^	0.58^∗∗^	-0.05	_			
(8) Self-kindness	-0.05	–0.14	0.39^∗∗^	0.64^∗∗^	-0.08	0.59^∗∗^	-0.08	_		
(9) Over-identified	0.32^∗∗^	0.43^∗∗^	-0.09	-0.54^∗∗^	0.53^∗∗^	0.17^∗^	0.59^∗∗^	0.02	_	
(10) Mindfulness	0.03	–0.18^∗∗^	0.54^∗∗^	0.64^∗∗^	0.03	0.58^∗∗^	-0.15^∗^	0.61^∗∗^	-0.04	_
*M*	9.49	25.82	31.16	3.22	2.73	3.24	2.78	3.21	2.96	3.34
*SD*	4.28	6.58	5.25	0.39	0.59	0.69	0.75	0.57	0.67	0.69

*^∗∗^p < 0.01, ^∗^p < 0.05.*

### Mediation Analysis

**FIGURE 1:**
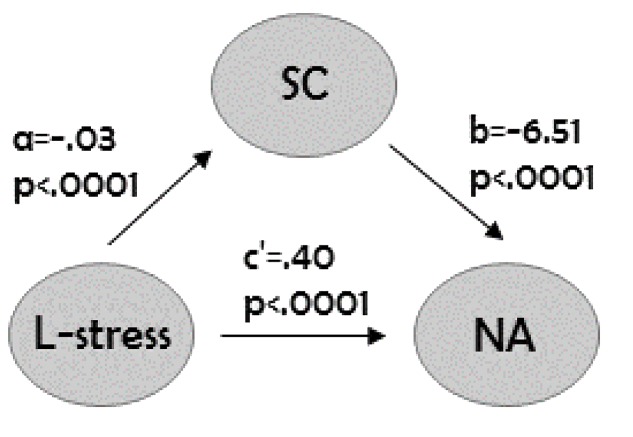
**Mediation analyses of self-compassion on the relationship between learning stress and negative affect.** Learning stress served as independent variable. Path *a* is the effect of learning stress on the proposed mediator, while path *b* represents the effect of the mediator on negative affect. Path *c’* shows learning stress’s direct effect on negative affect. L-stress, learning stress; SC, self-compassion; NA, negative affect. Result presented here is based on 5,000 bootstrap samples.

Results showed that self-compassion mediated the effect of learning stress on the negative affect, with the estimate of 0.17 and a 95% BCa bootstrap confidence interval (CI) of 0.08–0.28 (see **Table 2** and **Figure 1**). We can claim based on this result that the difference between the total and direct effect of self-compassion on the negative affect is different from zero. A high learning stress was associated with a low level of self-compassion that linked to a high negative affect.

**Table 2:** Mediation results presented based on 5,000 bootstrap resamples.

	Total effect	Direct effect	Path *a*	Path *b*	Indirect effect
Self-compassion	0.56^∗^^∗^	0.40^∗^^∗^	-0.03^∗^^∗^	-6.51^∗^^∗^	0.17 (0.08–0.28)^†^

*Adjusted coefficients with 95% confidence intervals. ^†^Indicates a 95% confidence interval that does not include 0. Learning stress served as independent variable.*

## Discussion

This study explored the protective role of self-compassion in a cohort of undergraduate students with chronic academic stress. The negative and positive relationships between the learning stress and subscales of self-compassion were identified in the present study. These results are expected because of the bipolar characteristics (i.e., positive and negative wording items) of the six components of self-compassion. The results also indicated that self-compassion negatively mediated the relationship between learning stress and negative affect. Taken together, these findings suggest the protective role of self-compassion to buffer chronic academic stress and its influence on emotional response (Leary et al., 2007; Neff et al., 2007a,b; Neff and Vonk, 2009), thereby further indicating that self-compassion-centered interventions can be developed to assist students in coping with academic stress.

Expectedly, a limited learning stress is associated with improved self-compassion, thereby resulting in limited negative affect. This result is evident in previous studies, which also determined that self-compassion could buffer an individual’s emotional response to chronic academic stress (Neff et al., 2005; Kyeong, 2013; Hope, 2014). Using a stressful sample, the present study extends the stress-buffering effect of self-compassion to a cohort of participants that report a significantly higher academic stress than their peers; this case has not been reported in existing studies. Allen and Leary (2010) proposed that the essential of self-compassion is to treat yourself kindly when facing adversities. Furthermore, self-compassion involves positive cognitive restructuring process, in which individuals could change their point of views of stressful events to determine the positive aspects behind these negative events. Therefore, the students with a high level of self-compassion would treat themselves kindly and with acceptable attitude under chronic academic stress. They would take PEE as a self-improvement and self-growth path. Even if they fail in this examination, they are still able to treat this matter in a positive manner. Studies have also shown that self-compassion could lead to increased mastery goals among undergraduate students through limited fear of failure and improved perceived competence (Neff et al., 2005). Accordingly, an individual who is high in self-compassion can cope well with stress; they may be able to stay calm in the face of academic stress. Thus, undergraduate students with high self-compassion may not be overwhelmed by learning stress and the coming examination. Therefore, they do not experience considerable negative affect as those with low self-compassion do.

Neff (2003b) considered self-compassion as a beneficial emotional regulations strategy. That is, negative emotions, psychological distress, and painful feelings are accepted in awareness with kindness, understanding, and non-judgmental attitude. Lazarus and Folkman (1984) distinguished between the problem- and emotion-focused coping strategies in the literature. Problem-focused coping manages or alters the problem that causes the distress, whereas emotion-focused coping aims to directly regulate emotional responses to a problem (Lazarus and Folkman, 1984). From this point of view, individuals with high self-compassion tend to adopt substantial emotional and adaptive coping responses to academic stress. Neff et al. (2005) reported that self-compassion is positively associated with emotionally adaptive coping responses (e.g., reinterpretation and acceptance) but negatively associated with maladaptive coping responses (e.g., denial and mental disengagement) among students who were recently confronted with an academic failure. The similar result was replicated in another study among first year master’s students (Ying and Han, 2009). Consequently, students with high self-compassion would like to transform negative cognition and emotions into substantially positive state, as well as further adopt actions to change themselves or the external environment in the appropriate and effective manner (Carver and Connor-Smith, 2010).

Brief self-compassion induction programs have been developed based on the preceding findings to modify the participants’ self-relevant thoughts, emotions, and behaviors (Adams and Leary, 2007; Leary et al., 2007). Adams and Leary (2007) asked female participants to think about overeating in a self-compassionate manner to analyze the relationship between eating guilt and self-compassion. The results indicated that the eaters who were given the self-compassion induction showed limited distress and ate less (Adams and Leary, 2007). Nevertheless, comprehensive interventions that rely on self-compassion are limited in the education context. Educators or educational psychologists can develop interventions that focus on positive cognitive restructuring to assist students positively view themselves and their situations with considerable self-directed compassion. Iskender (2011) demonstrated that self-compassion was significantly related to academic procrastination and dysfunctional attitudes. Accordingly, educational self-compassion induction programs may be a promising method to change these attitudes and correct behaviors. In addition, other interventions can be used to enhance the participants’ self-compassion. For example, mindfulness-based stress reduction programs have been determined to enhance self-compassion (Birnie et al., 2010). Thus, future studies should also explore approaches that enhance self-compassion through traditional and mature interventions. Recent studies also indicated that mindfulness may help individuals to clearly identify their character strengths, which further result in enhanced wellbeing (Duan, 2016a). Accordingly, the mechanisms underlying mindfulness and self-compassion have to be examined.

Despite these results, the present study presents a few limitations. Self-compassion exerted direct and indirect effects on stress in two female chronic illness groups, namely, inflammatory bowel disease and arthritis (Sirois et al., 2015). On the one hand, self-compassion reduced stress through the substantial use of adaptive coping (e.g., positive reframing, acceptance) and limited use of maladaptive coping (e.g., behavioral disengagement, self-blame). On the other hand, self-compassion was associated with low stress through routes beyond coping responses. Similarly, the current study experiences difficulty in concluding whether self-compassion reduces negative affect via modulating stress response directly or indirectly by coping with academic stress. This issue can be addressed in future studies with specific designs. Other limitations should also be mentioned. First, the gender factor is considered a control variable in the current study. Existing studies suggested that females tend to be considerably critical of themselves, which is associated with limited self-compassion (Neff, 2003b). Therefore, whether gender is a moderator in the self-compassion buffer models is unclear. Second, other studies implied the moderating role of self-compassion in the relationship between academic burnout and psychological health (Kyeong, 2013). Further studies are necessary to analyze or distinguish the mediation and moderation roles of self-compassion in the stress study areas. Third, other emotional outcomes such as well-established flourishing (Duan and Xie, 2016; Tang et al., 2016) and thriving (Duan et al., 2016) need to be adopted in future studies to explore the emotion-buffer effects of self-compassion. These comprehensive wellbeing indicators will deep our understanding of the role of self-compassion in stressful situations.

## Author Contributions

The study was designed and the data was collected by XL and XC. The manuscript was prepared by YZ, XL, XC, and WD. WD finalized the final submission.

## Conflict of Interest Statement

The authors declare that the research was conducted in the absence of any commercial or financial relationships that could be construed as a potential conflict of interest.
